# When the Masks Come Off in Canada and Guatemala: Will the Realities of Racism and Marginalization of Midwives Finally Be Addressed?

**DOI:** 10.3389/fsoc.2021.632053

**Published:** 2021-07-14

**Authors:** Betty-Anne Daviss, Tammy Roberts, Candace Leblanc, Iris Champet, Bernadette Betchi, Angela Ashawasegai, Laura Gamez

**Affiliations:** ^1^The Pauline Jewett Institute of Women's and Gender Studies, Faculty of Arts and Social Sciences, Carleton University, Ottawa, ON, Canada; ^2^l'Hôpital Montfort and The Ottawa Hospital, Midwifery Divisions, Ottawa, ON, Canada; ^3^Independent Researcher, Elliot Lake, ON, Canada; ^4^Independent Researcher, Ottawa, ON, Canada; ^5^Asociación para la Promoción, Investigación y Educación en salud en el Occidente de Guatemala (PIES), Quetzaltenango, Guatemala; ^6^Algonquin College, Ottawa, ON, Canada; ^7^Independent Researcher, Hamilton, ON, Canada

**Keywords:** Canada, COVID-19, Guatemala, IBPOC, racism, social justice, midwifery

## Abstract

This article addresses the effects of COVID-19 in Eastern and Northern Ontario, Canada, with a comparative glimpse at the small province of Totonicapán, Guatemala, with which Canadians have been involved in obstetric and midwifery care in particular over the last 5 years. With universal health care coverage since 1966 and well-integrated midwifery, Canada's system would be considered relatively well set up to deal with a disaster like COVID-19 compared to low resource countries like Guatemala or countries without universal health care insurance (like the USA). However, the epidemic has uncovered the fact that in Ontario, Indigenous, Black, and People of Color (IBPOC), as elsewhere, may have been hardest hit, often not by actually contracting COVID-19, but by suffering secondary consequences. While COVID-19 could be an issue through which health care professionals can come together, there are signs that the medical hierarchies in many hospitals in both Ontario and Totonicapán are taking advantage of COVID-19 to increase interventive measures in childbirth and reduce midwives' involvement in hospitals. Meanwhile, home births are on the rise in both jurisdictions. Stories from a Jamaican Muslim woman in Ottawa, an Indigenous midwifery practice in Northern Ontario, registered midwives in Eastern Ontario, and about the traditional midwives in Guatemala reveal similar as well as unique problems resulting from the lockdowns. While this article is not intended to constitute an exhaustive analysis of social justice and human rights issues in Canada and Guatemala, we do take this opportunity to demonstrate where COVID-19 has become a catalyst that challenges the standard narrative, exposing the old ruts and blind spots of inequality and discrimination that our hierarchies and inadequate data collection—until the epidemic—were managing to ignore. As health advocates, we see signs that this pandemic is resulting in more open debate, which we hope will last long after it is over in both our countries.

## Introduction: a Glimpse at Some of the Social Justice Issues in Ontario and TotonicapÁN, Guatemala

*I was up late dealing with two births, exhausted, and developed a fever of 38.1 Celsius (100.6 Fahrenheit). I knew that I would have to go to the local hockey arena—the setting designated as the COVID testing site in Ottawa, Canada. I thought, how great a thing to have it in an ice hockey arena, where everybody should feel at home because they are familiar with the setting. Indigenous, Asian, Somali, Lebanese, Punjabi, and White families alike can take advantage of our Rideau Canal when it freezes over in winter—“the longest skating rink in the world”*—*winding through and connecting various parts of the city and the many ice hockey arenas*.*It was a sight to behold. There at Brewer's Arena, everybody lined up like good sports, no ethnicity, race, class, profession, or gender being given priority—except kids, who, with their parents, were afforded a private entrance up to the bleachers where they got to wait until their turn came and to pretend that they were watching a game down on the rink*.

*The rink—bereft of ice*—*was now covered with us would-be “game contestants” sitting dutifully on the edges of our fold-up black chairs, all neatly lined up a hockey stick apart (i.e. 6 ft). After each of our OHIP*^1^ *cards had been checked, we had to give the history about why were there: we were phoned by a friend that they had COVID, we had traveled, or we had symptoms. As the “Head Coach” on the microphone called out our names, we were instructed politely to turn our chair around as we rose so that the chair cleaners could easily identify those needing to have any COVID cooties whisked away with disinfectant. From there we got to get our poke in the nose*.

*Yet while we were all treated equally during this testing process, I knew that we were not all going home to equitable housing. We were not all returning to secure jobs, the same ability to social distance or feed our kids and grandkids during this time. And aware that women of color are one of the hardest groups hit with COVID, especially those working in nursing homes, I thought that they were the ones who actually* should *be given priority in the lines. And then I began to google hockey and racism, and was struck by the trouble on our hockey rinks, where immigrants send their kids, knowing it will help them blend into the social fabric in small towns, Ottawa, and Montreal—but where they are subjected to racial slurs. Who knows but maybe one of those little boys or girls were reliving their first incident of racism in this very arena as they sat up there in the bleachers? Canadians may have a society that prides itself on trying to be fair, but COVID-19 is providing us ample opportunity to re-evaluate our concept of ourselves*.

    -Betty-Anne, reflections from the COVID testing arena, 2020

A comparison about pandemic effects between a province in a high resource country (Canada) and one in a low resource country (Guatemala) might seem unfair, like comparing apples with oranges. In 2017, maternal mortality in Guatemala was 95/100,000^2^ and in Canada was 10/100,000 births^3^. Guatemala has the sixth-highest rate of chronic malnutrition (stunting or low height-for-age) in the world—at 47% percent—with the prevalence reaching around 70% in Totonicapán^4^—the Guatemalan province we wanted to compare with the province of Ontario, Canada.

Despite these large differences, comparisons can expose the good and the bad of any health care system, and this article reveals some surprising similarities between our two countries. Both countries at least profess to have universal health care, but the Indigenous populations in both countries suffer more from malnutrition, poverty, education, and health care disparities than others. Canada boasts well-integrated midwifery services and complete government funding for birth at home, birth center, or hospital. Guatemala has midwifery services, but they are unsupported by the government. As in other countries, COVID-19 has exposed the inequities in the Canadian social/health and welfare system. In this article, we present an overview of how, with some success, we have dealt with COVID-19 in the province of Ontario, but also how it has negatively affected populations with health and economic disparities, in particular IBPOC^5^ people, forcing, among other measures, racial/ethnic data retrieval to become a priority.

This Introduction is providing an overview and will explain how we came up with our methodology for the comparison. A brief review of historical events demonstrating the prevalence of racism in both countries provides some context to understand what has led to inequities in the health care systems.

The section, The Effects of COVID-19: Which Populations Are Suffering Most? will compare COVD-19 cases and death rates in Canada and Guatemala and what ethnicities/races are most affected. It becomes evident that while racism has manifest as violent genocide and unbridled femicide in Guatemala, in Ontario, attempts to address subtle racism have been predominantly performative and symbolic.

Section Childbirth in Ontario under the Strain of COVID-19: Information Dissemination and Canadian Compliance explains the backdrop of compliance among Ontario citizens with COVID-19 followed by intimate first-hand accounts about the lockdown from:

1. a solo Registered midwife in Northern Ontario who describes the effect on her work among Indigenous communities, Amish and Mennonite communities (Section Northern Ontario Narrative);
2. midwives in Eastern Ontario who describe both increased collaboration and increased tension among the professions as a result of COVID-19 and
3. a Jamaican Muslim woman who describes an experience of subtle racism in hospital in Ottawa (Section How COVID-19 Has Affected Maternity Care In Eastern Ontario).

In the section, How COVID-19 Has Affected Maternity Care In Eastern Ontario data is presented on the socio economic status of Ontario midwife clients and how their choice for home birth increased quickly with the onset of COVID-19.

The section on Guatemala: Experiences Among the Traditional Midwives of Guatemala, provides narratives about the traditional Indigenous midwives' experience in Totonicapán, revealing similar as well as unique problems compared to Ontario midwives from the pandemic lockdowns.

We offer this work as a means to understand the problems and articulate how to improve those systems that are inherently racist, colonialist, sexist, white cisnormative, and biased toward a medical hegemony. Midwives, nurses, and physicians working with these populations often see (but do not always work on rectifying) the inequities, the biases, and the vulnerabilities. They also fall prey to trying to mitigate a system that can either become increasingly abusive or more forgiving during a pandemic—or both—as we will describe below.

### Methodology

This article was first conceived when a Vancouver, B.C. midwifery practice and Betty-Anne Daviss, a cisgender white midwife who began her midwifery career 45 years ago working with traditional midwives in Guatemala and now works in Ontario, jointly approached the National Aboriginal Council of Midwives (NACM) to solicit their engagement in a national article about COVID-19.

Betty-Anne also contacted the co-ordinators of the “Maternal, Newborn, and Child Health (MNCH) Project: Reducing Gaps for Indigenous Peoples in Totonicapán, Guatemala,” a project, which started in 2016 and will end in 2021, implemented by Horizons of Friendship (Horizons), a Canadian international development organization, and the Association for Health Promotion, Research and Education in the Western highlands of Guatemala (PIES de Occidente) working in Totonicapán. The exchanges between Betty-Anne and the staff at Horizons and PIES facilitated insights on how COVID-19 was affecting the people in the region^6^.

It was difficult to interview individual Maya K'iche' traditional midwives—called *comadronas*—in a “department” (province/state) where Spanish is the second language, where the high school literacy rate is 17.6%^7^, and where internet access is intermittent or limited. Some comadronas have email but the answers are not always forthcoming, or are very short. We decided to use the synopsis of what is happening in Totonicapán from the coordinator of the MNCH project at PIES, Dr. Iris Champet. Laura Gamez, who currently manages the Horizons of Friendship' MNCH project and has worked in other programs in Central America and Mexico, on conflict resolution, peacebuilding, policy development, and the delivery of humanitarian aid for forcibly displaced populations, provided invaluable commentary and edits on the situation in Guatemala.

While NACM thought the project important, their board cautioned that they did “not have the capacity to research and write a piece at this time. Indigenous midwives should be the ones doing this research and writing, from their own perspectives, but we cannot do this now.” Soon after, the Vancouver midwives dropped out of the project because of difficulties in accommodating to COVID-19 life/work challenges with their young families.

The focus was then narrowed to address the effects of COVID-19 in Eastern and Northern Ontario, Canada, to compare it with the department (province) of Totonicapán, Guatemala. Betty-Anne sent out an email with open and closed questions, to all midwives in the Eastern region of Ontario (approximately 50), about how COVID-19 has affected their practice and their clients. At first she received little response, she assumed, because of all the extra COVID-19 updates, complicated briefs, new rules, multiple types of PPE to try, and the feeling that danger was lurking everywhere, and the midwives were “COVIDed-out.” But the answers of the three who did respond were very enlightening, and she had other means through which to pry—occasional face-to-face workshops and online meetings, with follow-up clarifications via email.

Two Indigenous midwife practices in Eastern Ontario and three in Northern Ontario were approached. One Indigenous practice in each of the regions immediately responded with interest, one saying they would approach Six Nations as well to offer the story from their viewpoint about what was transpiring. A sign of the times, only one came through—Tammy Roberts, a midwife working with four Indigenous communities as well as non-Indigenous communities around Elliot Lake.

**Figure 1:**
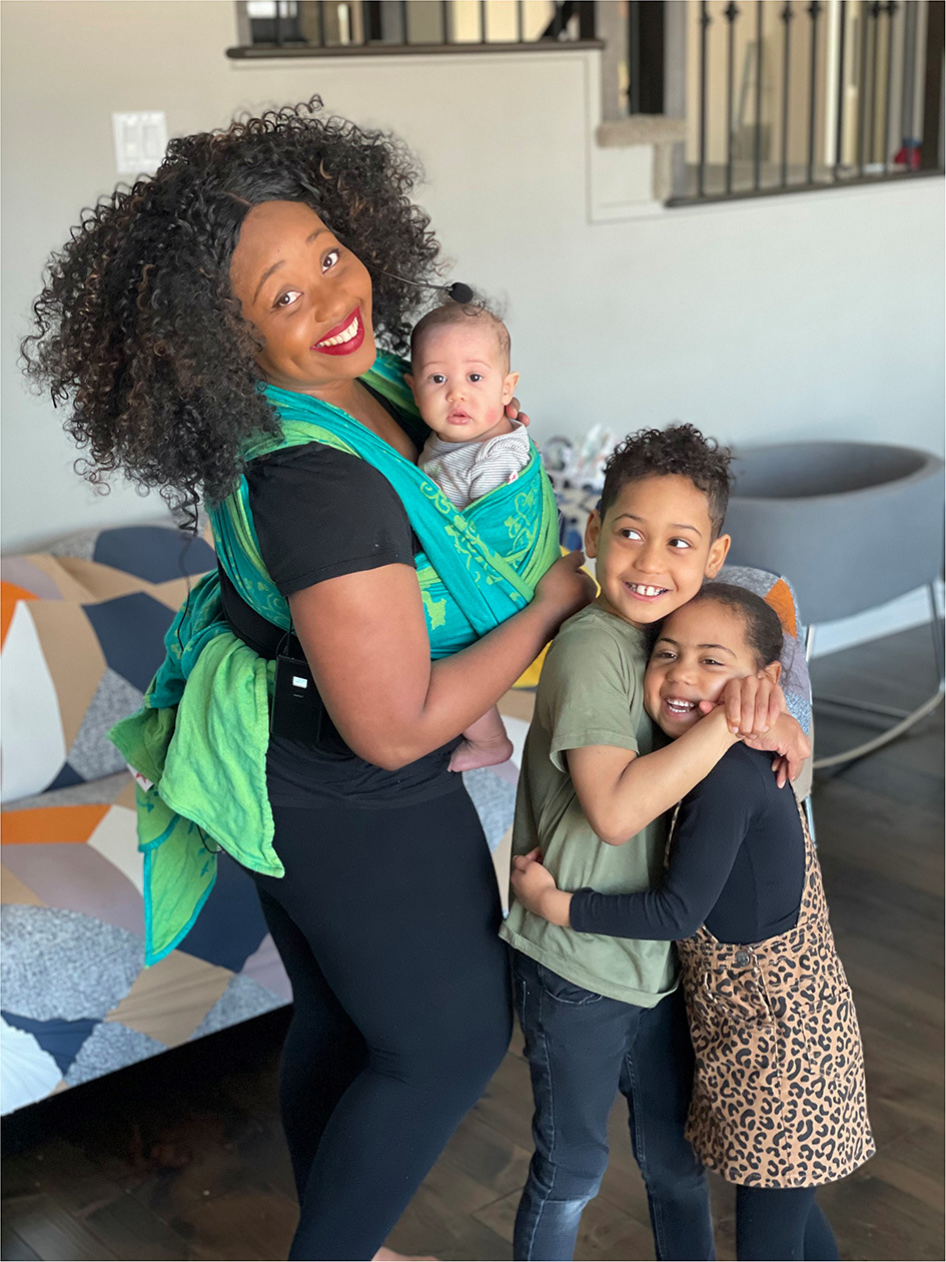
Bernadette Betchi recently joined in the Black Class Action Lawsuit in the Federal Court of Canada. She is employed by the Canadian Human Rights Commission. Her career with the Public Service began at the Canada Revenue Agency. “My experience working there was emotionally and physically draining. I moved from team to team, was bridged in as a term employee, while my white counterparts were all given permanent and higher positions right away.” She is seeking concrete, permanent solutions to undo the damage that has already been done, but looking ahead, for her children and their children, so they don't ever have to go through what her family is going through.

Betty-Anne then approached two midwifery clients who are activists in the IBPOC community: Candace Leblanc, who has been both a doula and a La Leche League Leader in Ottawa; and Bernadette Betchi, born in Cameroon, who grew up in Canada, worked as Sophie Gregoire Trudeau's Communications specialist and press secretary and then chose to leave for a job with the Human Rights Commission of Canada (see Figure 1). Both were instrumental in helping the article take shape with their perspectives as members of the IBPOC community.

We sought data regarding disparities among socio-economic groups and ethnicities and to study whether choice of birth settings changed following the pandemic. We solicited narratives from both clients and midwives to bring forward concerns and experiences navigating the pandemic—stories that cannot be told through any database. The questionnaire for both Indigenous and white settler practices asked:

1. Whether or not they had had any COVID-19 cases and how those were dealt with, clinically, logistically, and emotionally; what the compliance of clients and midwives was to the use of PPE (personal protective equipment) and lockdown procedures.
2. Whether dealing with the constraints of COVID-19 may have improved relations and unified forces at the hospital but also how the situation may have laid bare the limitations of the health care infrastructure.
3. Whether COVID-19 changes had improved or exacerbated structural inequalities for marginalized communities.
4. What other experiences indicated strengths or weaknesses, in the model of care around childbirth in their jurisdiction.

### Ontario, Canada: Some Good Health and Social Safety Nets Yet Colonialism and Racism Still Intact

Canadians have generally prided themselves on using government in positive ways. In fact, Canadians of all ethnicities have generally come to expect their government to ensure basic social programs—universal health care, unemployment insurance, education, social assistance, and human rights. Even if racism abounds, many Canadians are programmed at least to *believe* that everyone deserves to have the resources they need.

**Figure 2:**
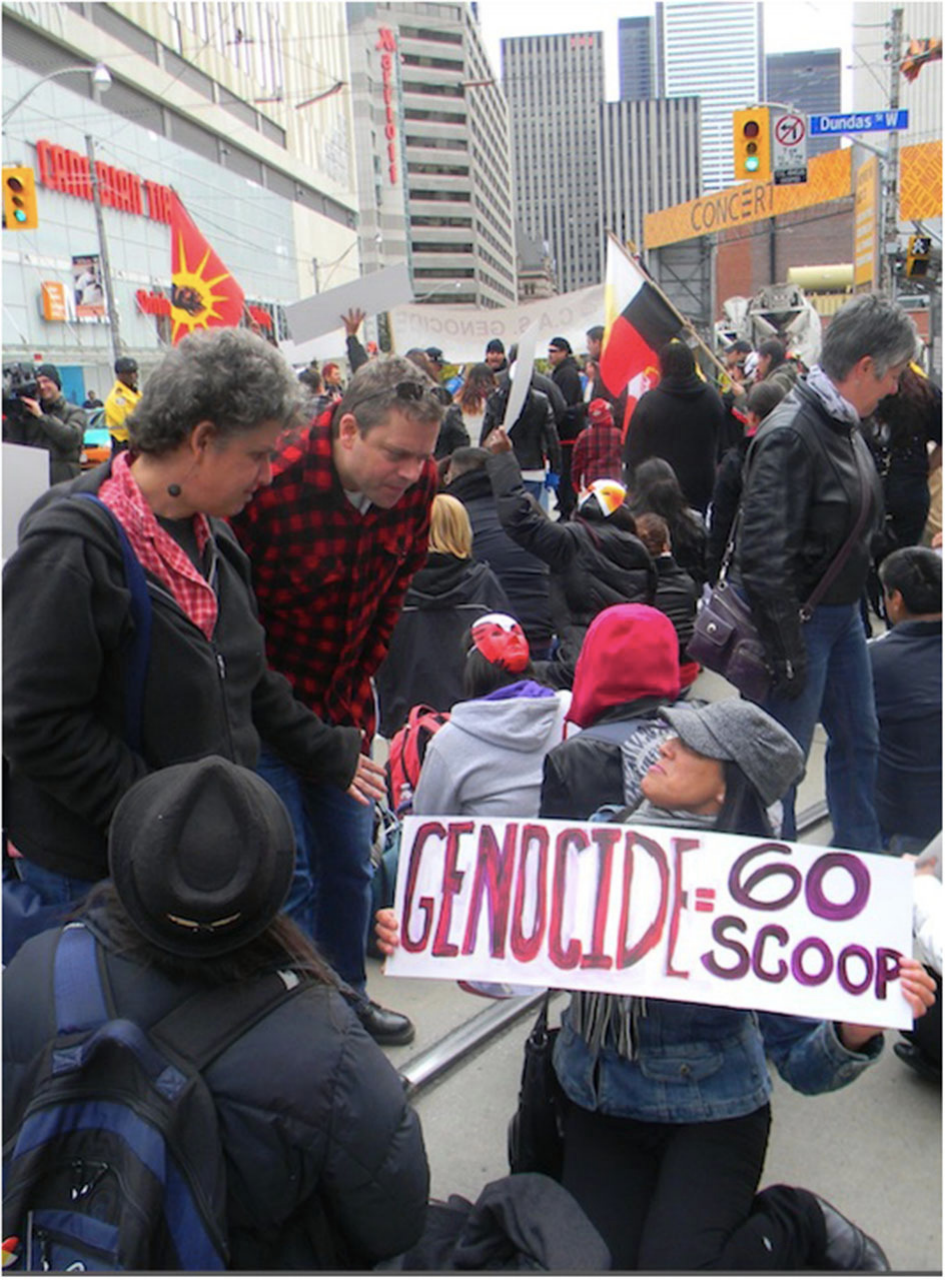
Two sympathetic passers-by stop to talk with 60s Scoop survivor, Angela Ashawasegai, at a rally in Toronto in 2011. Like most Canadians, they had never heard of the 60's Scoop or of the historical abuses of Indigenous children at the hands of White adoption and foster families until rallies and news media started drawing attention to it. Angela is now a certified trauma therapist, specializing in 60's Scoop trauma & Complex PTSD.

Because social programs express guarantees of human rights and commitments by governments to redistribute resources and to intervene in the market and the family to create equality, we are criticized by our US neighbors for harboring “socialism,” a branding which, hopefully, most of us wear proudly. However, over the last 15 years, a series of declarations and national truth commissions have exposed and shaken our foundation of pride in our system, exposing white privilege in Canada, starting first with regard to Indigenous rights:

1. The United Nations Declaration on the Rights of Indigenous Peoples (UNDRIP, United Nations Press Briefing, 1999) was adopted by the UN on September 13, 2007 to enshrine the rights that “constitute the minimum standards for the survival, dignity and well-being of the indigenous peoples,” which Canada shamefully at first did not agree to endorse.
2. The Canadian Truth and Reconciliation Commission (TRC) exposed the crimes of the Fathers of Confederation in establishing the residential school system, now acknowledged as a “cultural genocide” agenda to strategically take over Indigenous lands by eliminating Indigenous peoples' governments, language, and culture, by “killing the Indian in the child” in these schools (Truth and Reconciliation Commission in Canada, 2015, p. 1).
3. The Final Report of the National Inquiry into Missing and Murdered Indigenous Women and Girls (MMIWG) revealed that persistent and deliberate human and Indigenous rights violations and abuses are the root causes of Canada's staggering rates of violence against Indigenous women, girls and 2SLGBTQQIA people^8^.
4. The Sixties Scoop Settlement Agreement of 2018 was the result of winning a 9-year court case brought against the federal government by Indigenous people who suffered the loss of cultural identity when placed in white adoption and foster homes starting in the 1950s (https://sixtiesscoopsettlement.info). The principle of the financial settlement is a step in the right direction to begin the healing journey for their loss of cultural identity. But in the words of one of our authors, Angela Ashawesegai, it goes deeper: “I want closure for the historical abuse trauma. I was a child household slave and abused mentally, physically, and/or sexually on a daily basis. I'm still living with the haunting psychological impacts. We've only gone halfway with reconciliation with Canada.” (see upcoming book Lost Between Two Worlds: A 60's Scoop Adoptee's Search for Belonging forthcoming, 2020) (see Figure 2).

These events have somewhat shifted Canadian cultural understandings in a positive way toward expressed common goals. Since they transpired, grants for Indigenous midwifery in Canada have increased and a small but important exchange with Guatemalan health care providers included acknowledgment and respect for their traditional midwives because of their Indigenous rights. The latter is a milestone, as it is in direct opposition to the WHO/FIGO/ICM^9^ decision to instead stop the funding of traditional midwives, with little consideration of the negative effects on their communities (WHO, 2004).

Meanwhile, Black Lives Matter and other groups are finally gaining more attention to their persistent message of many years—that racism is very much alive in Canada, just as in the US, yet not the national priority it is becoming there (DasGupta et al., 2020; Desmond, 2020).

One of our authors, Bernadette Betchi, made a decision, along with over 600 other Black federal employees, that the time was rife in 2020 to finally launch a class action suit–long time coming– against the various federal departments in which they work^10^. They want to draw attention to the racism they find evident in so many parts of their lives, with intersections not even considered, starting with the very institutions that should be safeguarding human rights (see Figure 1).

Racism is rooted in a system that has been intentionally created in a way to benefit a very specific demographic. While COVID-19 and the uprisings following the George Floyd and Breonna Taylor killings were not the reason to launch the class action suit, the confluence of these events created a world that is now watching and listening, even in Canada. We suggest that the work done thus far by all political parties has been predominantly performative and symbolic in Canada. It will take some serious changes and a shift of mindset to dismantle the system of oppressions in which we live and that benefit some Canadians more than others.

### Totonicapán, Guatemala: Following Genocide Attempts, Indigenous Groups Are Gaining Some Recognition of Their Own Systems and Values

The Mayan K'iche', Mam, Kakchiquel, Kekchi, and other Indigenous groups have become more politically successful since the widely publicized exposure of the genocide carried out on their peoples by a succession of national military governments supported by the US (United Nations Press Briefing, 1999). The 36-year civil war saw the genocide of more than 160,000 Indigenous people (Horizons, 2018). One report says that the Mayan Quiché, living in the departments of Quiché, Huehuetenango, and Totonicapan (the department we are studying), were the victims of 80% of the massacres, “the worst hit of all the indigenous groups in Guatemala during the war [and which] remains the most discriminated against because of its past” (Research Directorate, 1998). Another report says that Totonicapán itself was less affected than some of the other communities, priding itself on being able to strongly protect its ancestral rights^11^; 98% of its population identify as Indigenous (Guatemalan Census, 2008).

Besides the historical and ongoing discrimination and marginalization of the Mayan Kiche' people as a whole, as more than 100,000 Indigenous women were victims of mass rape and forced into sexual slavery for the military (Horizons, 2018), women face an additional risk *because they are women*. Guatemala has the third highest femicide rate^12^ in the world. In spite of the fact that Guatemala championed a decree about femicide in its constitution in 2008^13^, 685 women were assassinated in Guatemala in 2010, compared to 213 in 2000 (Nobel Women's Initiative, 2012). Between 2000 and 2019, more than 11,594 women were victims of violence^14^.

The Nobel Women's Initiative also says that more than 95% of crimes against women are never even investigated by authorities because of the “machismo” that prevails in Mexico, Honduras, and Guatemala. This has normalized the violence and provided excuses for it.

More than 80% of the Maya K'iche' live below the poverty line. A large percentage of the population also lacks access to basic services like healthcare, education, clean water, and sufficient food (Horizons, 2018). Protests over mining and hydroelectric projects, educational reform, and access to land and public utilities^15^, and evangelical Christians wanting to impose their religion on Indigenous Mayan tradition^16^, occur in tandem with increased community demand that the traditional midwives become acknowledged and receive at least a token financial contribution from their government (Daviss, 2021).

Following amnesty after the war, a universal health care system was established, at least on paper (Pena, 2013). However, it has been hard to guarantee due to limited government resources and other problems regarding access. Studies from foreign goodwill ambassadors continue to lament the fact that Indigenous peoples in Guatemala do not often access modern reproductive health care (Ishida et al., 2012). However, PIES de Occidente chooses not to limit the definition of “good care” to one exclusively viewed through a modern medical lens, since it is well-known that the medical approach to birth conducts unnecessary inductions, forceps, and cesareans at high cost financially and to women's bodies (Anderson et al., 2021). As a result, the Canadian/Guatemalan exchanges strategically emphasize that the Canadians have been invited to “share” how they attend births, while the comadronas have in turn shared with the foreign health care professionals what they have in their bag of interventions, including the use of herbs and sauna sweats. This has both validated and entrenched the importance of their cultural nuances in an effort to reduce any attempts by the Canadians to impose *their* cultural norms during knowledge exchanges.

### Contrasting Ontario and Totonicapán Midwives

Ontario and Totonicapán midwives share with midwives around the world, the oppression of a medical hegemony that is threatening normal birth (Daviss, 2021). As Ontario midwives are required to fulfill a quota for the number of homebirths they do, and Indigenous midwives in Canada do not always choose to work within the confines of the mainstream Colleges (regulatory bodies)^17^, thus exclusively and legally serving Indigenous communities out of hospital, all Ontario midwives share the home birth experience with their traditional midwife counterparts in Totonicapán.

Unlike traditional midwives, Registered Midwives (RMs) in Ontario remain the primary care providers in hospital, unless major intervention like forceps or cesareans are necessary, when they transport from a home birth because they all have hospital privileges. Accommodations are also being worked out for non-registered Indigenous midwives in Ontario^18^. Prior to COVID-19, the traditional Indigenous midwives in Totonicapán were also permitted to attend their clients at the hospital and catch the baby in the birth position of the mother's choice. The PIES program implemented this acceptance of comadronas into the hospital to encourage, rather than discourage, them from bringing birthing persons to hospital if circumstances required medical surveillance or intervention. This program has demonstrated better collaboration, when supportive physicians admit that the traditional midwives have something to offer and treat them as part of their team.

## The Effects of Covid-19: Which Populations Are Suffering Most?

### Comparison of Cases and Deaths

**Figure 3:**
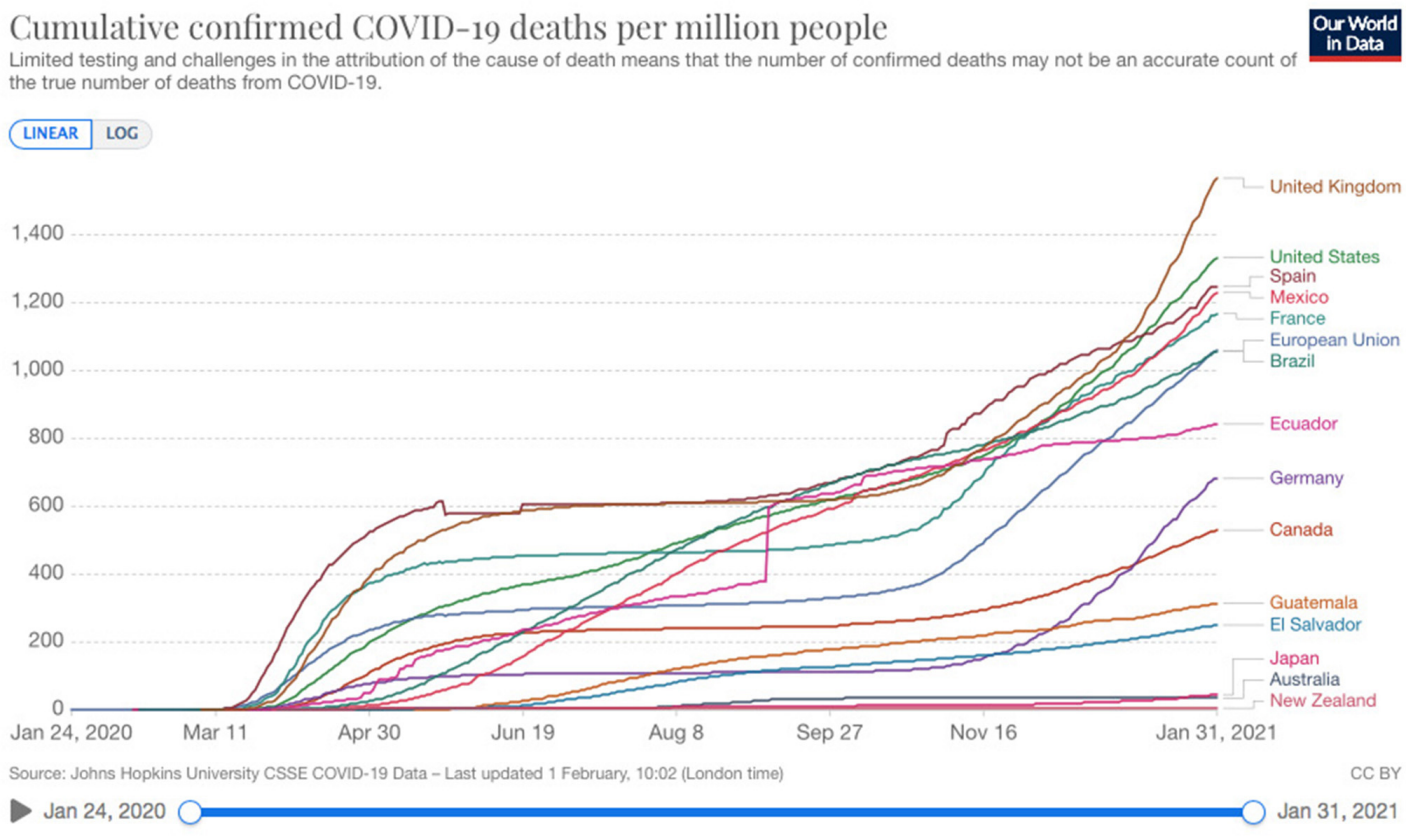
Our World in Data from the European Center for Disease Control January 31, 2021; https://www.ecdc.europa.eu/en.

As is seen in Figure 3 from the European Center for Disease Control, both Canada and Guatemala have boasted relatively low to moderate figures with respect to confirmed deaths from COVID-19 per million people. The approximate number of deaths per million in Guatemala at this time was under 400 and in Canada, under 600 per million. After a relative “leveling of the curve” that started in June 2020, by late September 2020, Canada was beginning to see a rise again in deaths per million people, while the numbers of cases in Guatemala have just continued to steadily rise.

There are multiple reasons why there is a relatively moderate number of cases in the two countries of Guatemala and Canada, but a fascinating one may be explained through the work of cultural psychologist Michele Gelfand, an expert on “tightness–looseness theory,” which explains variations in the strength of social norms and punishments across human groups (Gelfand et al., 2011). Her research indicates that “individuals in tight societies are more prevention focused, have higher self-regulation strength and have higher needs for order and self-monitoring abilities than individuals in loose societies.” They help people “to adapt to the level of constraint, or latitude, in their cultural context, at the same time, reinforce it^19^.”

The United States is described as having a “loose cultures” (Gelfand et al., 2011; Gelfand, 2021) and Mexico is described as tight in 2011 but by 2021 as “loose.” The category suggests interesting consequences:

*people in loose cultures had far less fear of the Covid-19 virus throughout 2020, even as cases skyrocketed. In tight nations, 70% of people were very scared of catching the virus. In loose cultures, only 49% were. Reality never bit in these populations in part because people in cultures that are adapted to low levels of danger didn't respond as swiftly to the “threat signal” embodied by the pandemic when it came (Gelfand*, *2021**)*.

Notice in Figure 3 which of these countries has more deaths per million people. Mexico and the US sit high on the graph, with their respective neighbors, Guatemala and Canada, far lower for the number of deaths reported per million. Although not assessed by Gelfand, it appears that the Guatemalan and Canadian cultures have some commonalities around being more “tight”—Guatemala possibly because it has learned to live a protective life following a 36-year civil war, Canada because it has a moderate culture which has had to develop community to withstand cold temperatures and its imposing American neighbor. One of the jokes describing polite, compliant, Canadian culture is, “How do you get 50 people out of the pool?” Answer: “Just say, ‘Everybody out of the pool.”'

Table 1 takes a point in time in September 2020 to compare the effects of COVID-19 on the two less-loose countries.

**Table 1:** Guatemalan and Canadian COVID-19 data September 9, 2020.

**Data obtained from national reports**	
Guatemala	Canada
79,622 accumulated registered cases*	134,294 accumulated registered cases^**^
68,308 estimated cases that have recuperated^*^	118, 000 that have recuperated^***^
2,897 cases that failed to register^*^	Not available
472.3 cases per 100,000 inhabitants^*^	357.27 cases per 100, 000 people^**^
2,897 deaths	9,223 deaths
17.2 died per 100,000 inhabitants^*^	24.47 per 100,000 inhabitants^**^
Life expectancy 2018, 74.06^****^	Life expectancy 2018, 81.95^****^
COVID-19 cases started slowly in March, grew in July, and continued to rise.	Cases started in March, with the first death March 9. Cases peaked by the beginning of May, and leveled off by the beginning of July, but a rise started in late September as schools began to open and people were becoming less vigilant.
Cases in the “Departmento” (province) of Totonicapan^*^	Cases in the province of Ontario
874/100,000 people, 8^th^ in place for number of cases, but 4^th^ for number of deaths.	301/100,000, while neighboring Quebec is at 757.15/100,000 people
Dependent on testing; it is not clear how well that is being done.	By September, testing was ramped up.

* *Ministerio de Salud Pública y Asistencia Social (2020)*.

** *Canadian Broadcasting Company (CBC) (2020)*.

*** *Corona Disease Canada (2020)*.

**** *World Bank (2018)*.

*~Laura Gamez at Horizons states: “Totonicapan up to October was in red alert as one of the highest case counts in the country with a case fatality rate of 6.4, almost double the national rate. The Guatemalan national registry of persons has seen an increase of deaths across the country, though most are not categorized as COVID-19 because they did not get tested. Dr. Iris Champet in communication with Horizons has shared that in many cases, COVID testing is not available in the communities; likewise, many community members do not get tested since there is a stigma surrounding testing positive. Only cases that have extreme complications and which are referred to the hospital are reflected here”.

Questions always arise about outcome accuracies in a country with low resources for data collection such as Guatemala. It is difficult to trust census data in a country with rugged mountain terrain and with Indigenous peoples of various dialects, not all of whom register their babies' births. But at least in Totonicapán, groups like PIES, for which physician Iris Champet is coordinating the MNCH project, are becoming more serious about accurately collecting and understanding the data, working in partnership with Indigenous midwives, and supporting their advocacy efforts to be officially recognized and paid a living wage (Daviss, 2021).

Considering the reservations from Laura Gamez and Betty-Anne's own thesis from the 1980s on Guatemala, namely that getting demographics in Guatemala is extremely difficult (Daviss, 1981), the available data on Guatemala may not be correct. The data suggest that in Canada we appear to have fewer cases per 100,000 of COVID-19 (472.3 cases in Guatemala vs. 357.27 in Canada) but continue to have more deaths per million people (17. 2 in Guatemala vs. 24.47 in Canada).

If the numbers are close to accurate, why would a high resource country have more deaths than a low resource country? Heart researchers suggest that Canada has seen higher COVID-19 deaths than many countries with fewer health care resources because more Canadians live longer with chronic heart disease, putting them at greater risk of dying from COVID-19 (Botley et al., 2020; Szklarski, 2020). In other words, because the Canadian health care system is pretty good and keeps people alive longer, it is the ones already sick or 65 or older who have been provided with good health care—the ones who might have already died in under-resourced nations like Guatemala before COVID-19—who are now dying instead in Canada as a result of COVID-19. Canada certainly has a higher life expectancy rate in non-pandemic times compared to Guatemala (see Table 1).

### Effects of COVID-19 on Indigenous Communities and People of Different Ethnicities

It is not clear how much more Indigenous populations are being affected by COVID-19 compared to white populations or Asian ancestries in Ontario. The narrative in Section Northern Ontario Narrative is set in the Algoma District in Northern Ontario, where about 20% of the population characterize themselves as Aboriginal (Cuddy and Moazzami, 2017), and data is very exacting as to how many fall into Metis, Inuit, First Nations, one, or all those categories (Statistics Canada, 2016). Then there is a weekly count of how many COVID cases are recorded at the Algoma health department^20^. But these databases do not connect and COVID-19 cases do not appear to be categorized by ethnicity—yet.

Another tactic in Ontario to find out who has been affected has been to try to obtain data from neighborhoods with high and low levels of “ethnic concentration” achieved through the Canadian census. Public Health Ontario has found that “ethno-culturally diverse neighborhoods in Ontario, primarily those concentrated in large urban areas, are experiencing disproportionately higher rates of COVID-19 and related deaths compared to neighborhoods that are the less diverse^21^.”

This difficulty in locating data on ethnicities is similar in Guatemala. It is clear, however, that the Guatemalan Health Ministry in 2017 found that the Guatemalan government was spending less per capita on health services in largely Indigenous departments than in departments with a majority of non-Indigenous communities^22^. This means that these rural areas are not prepared for the protection, prevention, and treatment that are required for COVID-19. Note that in Totonicapán, our comparison province, there were by September nearly double the cases per 100,000 than in the rest of Guatemala (see Table 1). Totonicapán was 8^th^ in line in the departments for number of cases of COVID-19, but 4^th^ in line for the number of deaths—and almost triple the cases of Ontario. It is possible that this is a result of the fact that Indigenous people constitute 98% of the population, with clean water, health care, and even PPE resources more difficult to come by.

With regard to Indigenous women in Canada, results of a survey and two consultations done by the Native Women's Association have revealed a spike in the number of Indigenous women facing more violent incidents since the pandemic began, suggesting that “more of these women are concerned about domestic violence in the midst of this pandemic than they are about the virus” (Wright, 2020). In Guatemala, it is understood that while COVID-19 exacerbates the problem of violence, presently there are suspicions that Guatemalan women are too afraid to call police on partners with whom they are locked down (Erum, 2020).

### The Elephant in the Room in Ontario

#### Bernadette Betchi and Betty-Anne Daviss

While health care professionals and the media in Ontario have been concentrating on specific groups at risk based on age and co-morbidities, a large Elephant in the room was asking in April, 2020, why, when Black Americans continue to experience the highest COVID-19 mortality rates in the US—more than twice as high as the rate for whites and Asians, who have the lowest actual rates (American Public Media (APM) Lab, 2020)—the Canadian government was not even collecting such data (Nasser, 2020)? Data reports cannot report what they do not collect. Yet by June 2020, with racial focus becoming mandatory because of uprisings in the US, Ontario changed course and now mandates data collection around race, income, household size and language when following up with people infected with COVID-19 (Farooqui, 2020). We still see little on the radar about 2SLGBTQQIA people.

No doubt concerned by the void in knowledge of racial disparities in Canada, a partnership between the African-Canadian Civic Engagement Council (ACCEC) and the Innovative Research Group (INNOVATIVE) produced a report from a survey posted on their website (ACCEC INNOVATIVE, 2020). It revealed that:

- Black Canadians are more likely to report COVID-19 symptoms, in either themselves or someone they know, more likely to say they sought treatment for COVID-19, and nearly three times as likely (21 to 8%) to report knowing someone who has died due to the virus.
- Black Canadians are more likely to report that their job requires them to work with people face-to-face (Net: +41 vs. +25 national average).
- Black Canadians are more likely to feel that no matter what steps they take, their day-to-day routine puts them at an uncomfortably high risk of catching the virus (Net: −2 vs. −17% national average).
- Black commuters are much more likely than the national average to report symptoms, to seek medical treatment, and to admit themselves or know someone admitted to the hospital, and twice as likely as average Canadian workers to say their commute is unsafe (24 vs. 12%). Black Canadians who commute to their work are also twice as likely to use public transit as other Canadians (25 vs. 12%).
- Black Canadians report much worse financial impact from COVID-19 than other Canadians (2020).

One reporter used the continually updated ECDC graph (Figure 3) to calculate during the first week of September that “If the US had Canada's Covid-19 death rate, 100,000 more Americans would likely be alive today” (Lopez, 2020). However, comparing ourselves to the country that has the most cases in the world gives Canadians a superficial sense of satisfaction that perpetuates the myth that we care more about our marginalized and racialized communities. This false sense of superiority has been maintained and perpetuated for so long that Canadians have a difficult time analyzing their history and acknowledging their faults. Although Canadians have universal health care insurance, they can't, like US citizens, access National Health Institute data to understand where their disparities lie.

In sum, until January 2021, we see that the approximate number of deaths per million in Guatemala is under 400 and in Canadians under 600 per million. It is difficult to obtain data on Indigenous groups in either jurisdiction but it is suspected that at least in Totonicapán, the Indigenous population is at increased risk both from COVID-19 as well as violence. In Ontario, it is clear that communities that have more ethno-culturally diverse populations have increased number of COVID-19 cases and two NGOs have demonstrated that the Black population is at increased risk.

## Childbirth in Ontario Under the Strain of Covid-19: Information Dissemination and Canadian Compliance

The “tighter culture” and pool analogy may explain why Canadians are relatively compliant, and COVID-19 restrictions were met with obedience, especially at the beginning during the COVID lockdown.

Like everyone else, midwives in Ontario were thrown into a difficult situation, with rules and regulations—best practices based on uncertain data—changing weekly, sometimes daily. The Provincial Council for Maternal and Child Health (PCMCH) was tasked by the Ministry of Health to put together an expert group to address practice changes regarding maternal-neonatal health in relation to COVID-19^23^. It included representatives from nursing, midwifery, obstetrics, family practice, pediatrics, neonatology, infectious diseases, and microbiology. They created guidelines and continue to update practitioners about best care. The committee recommended that obstetric interventions be based on obstetric indication rather than COVID-19 status. That is, if a blood pressure looks bad, or a baby is in distress during labor, appropriate interventions are used, but it is not assumed—as it has been in other countries—that inductions or cesareans are better for the mother or baby based on a COVID-19 diagnosis alone. In this respect, Ontario guidelines resonate with the recommendations of the International Federation of Gynecology and Obstetrics (FIGO Safe Motherhood and COVID-19, 2020).

Following this committee's recommendations, there was general agreement and understanding by mid-March in Ontario that only one support person would be permitted at hospital, birth center, and home births alike, as the home setting in Ontario is treated like any other institution. Most clients have been compliant with this restriction, in keeping with the community spirit of protecting the healthcare practitioner as well as the parents. While everybody wanted to have doulas and other friends and family attend their births, general accounts, in particular in the first months, suggest that clients did not feel overly oppressed by this limitation, knowing that many in our neighboring country (the US) were not permitted even to have their spouses attend.

## Northern Ontario Narrative

### Tammy Roberts

We respectfully begin the narratives with a candid reality piece from a Registered Midwife, Tammy Roberts, whose practice in a rural Indigenous community, like the others, is “locked down,” submitted August 31, 2020. Tammy Roberts has mixed Aboriginal/white background and grew up in Northern Ontario. She answered our questionnaire orally and then edited the transcript, focusing on issues of compliance and how her relations with the hospital and the community changed as a result of COVID-19 and the hoops she found most difficult to jump through.

The National Aboriginal Council of Midwives (NACM) would like us to clarify that the perspective being shared is that of the registered midwife. Indigenous Midwives are the only midwives not required to register with the College of Midwives to legally practice in Ontario; they are exempt as stated in the Midwifery Act. Tammy chose to become registered to serve both the Indigenous and non-Indigenous population in her community.

### The Lockdown in a Northern First Nations Community

I work as a midwife along the North Channel of Lake Huron. My catchment area includes four First Nations communities: Serpent River, Sagamok, Mississauga, and Thessalon. To date there have been no reported cases in these communities, and only three reported cases within my catchment area. The surrounding areas of Sudbury and the Sault have had just over 120 reported cases combined (This was written in August; cases shot up from 5 to 38, 3 days after Christmas).

In March, every First Nations community within my catchment imposed a lockdown, banning visitors and monitoring community members' travel. Each community dealt with things a little differently. One community initiated a pass system, allowing only one trip per week outside of the community. As the restrictions eased, it became twice a week. In other communities, people were encouraged, but not mandated, to limit travel.

In order to access the communities, I had to be added to the list of essential workers each community had developed. When I communicated with the Chief in one community, I was advised that their essential worker list was generated by community members identifying who would be attending at their residence, and I was instructed to have clients reach out to the person in charge of the essential worker list to have my name added. If a client doesn't want someone to know of the pregnancy, they can advise the list coordinator that they are receiving regular visits from an essential worker and that the Chief is “aware,” of the confidential situation.

Blockades were installed at each entrance to each community and access was allowed only at the main entrance. Yet the monitoring was inconsistent. Some communities had 24/7 monitoring; others had unmanned blockades but a police presence in the community monitoring for non-community members. For instance, when I arrived at the barricade of one very strict community, I had to identify myself as a midwife working in the community. They wanted to know whom I am visiting, but due to confidentiality I could not say. Referring to PHIPA (the Personal Health Information Protection Act), has worked to grant access.

One of my clients had a family member working at the Band office. The client had a history of precipitous birth and the family had some anxiety about whether I would arrive before the baby, once labor started. My client's family member took measures to ensure I would have quick access and be able to avoid the screening measures and the lineup at the blockade. On the day of the labor, things were progressing quickly as expected. The family member called and advised the border attendants that I was on my way and that I would just wave on my way by, which I did. I arrived at the client's home just in time to have a listen to the baby's heart rate and set up my equipment before the baby arrived!

### Compliance in My Community

All my prenatal and postpartum visits are done in clients homes. My clients and their families have been good about self-isolating. In March and April, I had clients who had been sent home from work with pay for as long as 3 weeks to wait things out and see if it was safe for them to return to work. Many have taken early maternity leave or sick leave. Highway traffic has been very sparse and in town, pedestrian and vehicle traffic also very minimal—proof that people have been taking the recommendation to stay home very seriously. The mask wearing rate in our immediate area is quite good; it is rare to see someone in public without a mask.

The Amish and Mennonite communities that I serve received visits from public health personnel with updates on the guidelines. Initially they were permitted to continue holding church services, but as the allowable numbers for gatherings diminished, church services were eventually discontinued. Once the numbers for gatherings increased again, church services resumed, sometimes in creative ways such as outdoor services held in fields, with each family staying in their own buggy to align with physical distancing guidelines. Due to the limited number of school-aged children in one community, they were able to continue holding classes until the numbers for gatherings fell below ten. Most of the young children were from the same family, so they were allowed to continue having classes. For the older children from other families, the teacher would drop lessons off weekly at the house.

The Chief of Obstetrics for St. Joseph's General Hospital in Elliot Lake became the COVID point person for our area. She stayed abreast of the most current guidelines and research and disseminated that information throughout the area. We have a radio station and formal and informal online news sources, which she contributed to regularly.

### Did Relations Change at the Hospital?

Relations have indeed changed at St. Joseph's General Hospital in Elliot Lake since COVID struck. Mostly, existing relationships were reinforced, but our Chief of Obstetrics was very keen to limit unnecessary hospital visits. Early discharge was actively encouraged, and home birth was recognized as a good option. As things progressed, issues I would normally consult for in person or send somebody into the hospital for, like postpartum hypertension, were dealt with outside of hospital over the telephone if possible and reasonable. I give the history, prescriptions are sent to the pharmacy, and I follow up with the client as per the physician's advice, limiting hospital visits and personal contact.

Our COVID assessment is quite unique. The Family Health Team swabs people at their home. If a swab is required, a nurse calls to advise when she is on her way, the patient waits at the front door, the nurse dons her PPE at her vehicle, and then performs the swab. She had an assistant to help with donning, doffing, and sanitizing.

Blind River is about 40 min from Elliot Lake. Normally clients in that area of the catchment will have outpatient lab work and ultrasound done in Blind River; however, all non-urgent investigations have been postponed indefinitely. The definition for “urgent” is very limited and does not apply to routine pregnancy, so even clients needing repeat screens and RhIg (Rh Immune globulin) have traveled to Elliot Lake rather than having it done in their own community. Transportation is a challenge for many people in our area, so that has added hardship for some.

### Biggest Problems: Isolation and Endless Emails

As a solo midwife, with only a part-time administrator, one of my biggest challenges has been keeping up with the volume of COVID-related information. With the degree of concern related to COVID, I felt that I had to stay on top of all of the emerging information and recommendations. That meant reading endless e-mails, watching the news, and checking public health websites. Add in the administrative work of sourcing PPE and mandated responses to surveys regarding PPE stock and usage—it is all quite onerous for one person. Making contingency plans for who would take over should I get COVID-19 and just getting help to cover myself once in a while to get rest has been onerous.

### Summary of the North Channel

In Tammy's account, we observe the brutal reality of living a life as an isolated midwife in a rural area without backup, worsened by the pandemic. There is evidence of compliance with PPE, as is in keeping with the Canadian “tight” culture. At least for the most part, this rural midwife feels supported by those whom she serves, the hospital, and the community border patrol. She feels her credentials and the confidentiality of her clients are respected. Her main problem is her feeling of isolation as a single midwife in a practice in a province where urban practices boast 8–20 midwives, with one or two people able to take the load of figuring out where to find the PPE, how to put it on, where to keep it, how to keep the clinics safe, and how to follow each new recommendation. To examine how other midwives reacted to pandemic regulations, in Ontario we turn to narratives from Eastern Ontario.

## How Covid-19 Has Affected Maternity Care in Eastern Ontario

### Betty-Anne Daviss

When I became engaged in this article, I saw COVID-19 in our hospital as a juncture that was offering an important unifying issue through which all parties were coming together to sort out the best ethical care for childbearers. I watched with great relief at how well physicians, nurses, and midwives rallied around the COVID-19 issue at the Montfort Hospital.

I sent out the survey to 50 midwives, being particularly interested in knowing whether or not my hunch was correct and other midwives were feeling the same way I did about COVID-19 being a *boost* to relations among hospital staff, but also about how they were feeling it was affecting clients.

### Compliance

At first not all midwives and nurses providing frontline care were provided with masks, due to supply demands. Midwives ended up being supplied with bonnets, drapes, and masks homemade by clients, dentists and dental hygienists, and even by Tim Horton's (a fast-food restaurant chain). Visors were made by an innovative midwife with a 3-D printer. Following provincial recommendations, by May 2020, all mothers in Ontario hospitals were required to wear masks even in pushing stage, but this was not enforced with all births outside the hospital in our area (at home birth and the Ottawa Birth and Wellness Center), as mask wearing during pushing makes it much harder to breathe. While most clients have adopted to not having their spouses at visits, but permitted to at least have them at the birth, clients in our area have threatened to birth unassisted, and some have done so because of the restrictions both in hospitals and in birth centers regarding the number of support people allowed. Two practices have reported that even some who are at higher obstetric risk, yet fear the virus or the restrictions more, have given birth unassisted. While those numbers are not determinable, preliminary data suggested an increase in home births among the midwives (see Table 2).

**Table 2:** Planned place of birth for Ontario Midwifery Clients, March to May, 2019 and 2020.

**Month and Year**	**Hospital**	**Home**	**Birth Center**	**Midwifery Clinic**	**Total**
March 2019	1517	260	86	14	1877
	(80.8%)	(13.9%)	(4.6%)	(.7%)	(100%)
March 2020	1073	173	79	19	1344
	(79.8%)	(12.9%)	(5.9%)	(1.4%)	(100%)
April 2019	1555	245	82	17	1899
	(81.9%)	(12.9%)	(4.3%)	(0.9%)	(100%)
April 2020	1028	235	51	29	1343
	(76.5%)	(17.5%)	(3.8%)	(2.2%)	(100%)
May 2019	1509	266	106	16	1897
	(79.5%)	(14.0%)	(5.6%)	(0.8%)	(100%)
May 2020	934	252	48	21	1255
	(74.4%)	(20.0%)	(3.8%)	(1.7%)	(100%)

### How Relationships Have Been Affected at the Hospitals in Eastern Ontario

At the Montfort Hospital, my home hospital, the difficult tight rope in protecting the baby from the mother who has tested COVID-19 positive, yet still affording the baby breast milk and maternal/infant bonding, were sorted out based on informed choice and safety standards. It has been established among pediatrics and the obstetric/nursing/midwifery team, based on the Task Force recommendations, which did not—like the CDC in the US, and in other jurisdictions like China and India—recommend that COVID+ mothers be separated from their babies.

I was awestruck at how thoughtful the letter was from our Head of Pediatrics to all staff at our hospital:

Parents have the legal and ethical right to make these decisions for their babies. At NO time do we have the right to remove a baby from its parents unless we have a legal order from the CAS [Children'Aid Society], or if ethically the health care professional is concerned for the baby's well-being with the decisions made by the parent….in our recommendations to parents who are either suspects or COVID positive, it is very important to present the facts and known risks to their newborn baby so far. Since we have no evidence of harm to the baby if the mother wishes skin-to-skin or to breastfeed (with the precautions mentioned in the pandemic plan)**, we cannot refuse this**.

We cannot separate the baby from the mother without her consent (again implicitly if the baby is not sick after birth).If the baby needs admission to NICU care, he will be separated from mom—like other non-COVID-19 babies who require that care. The difference now is the protection of the unit and our staff, as well as other patients. Parents cannot visit their babies at NICU if they are suspected or positive for COVID-19—as this puts the well-being of many people at risk.It is important to continue to practice with empathy, because what these parents are going through is also extremely difficult for them.

-Julie Nault, Head of Pediatrics, Montfort Hospital, April 24, 2020

This is only part of Dr. Nault's statement, but paragraph after paragraph were laid out passionately, delicately, clearly, and on high moral ground. The context is that we had been through several years of hostile meetings over whether and how to attend breech births (see Daviss and Bisits, 2021) and over issues around whether or not midwives should be consulting for labor induction. Informed choice, on which our midwifery profession is based, has been a hard sell at times with the other health professionals at the hospital, who do not always think our clients make wise choices or that we bring them to their senses enough to follow the rules. But when it came to COVID-19, the Head of Pediatrics stated what we could all be on board with.

In other hospitals, however, after only three midwives answered the initial email request, negative comments began to trickle in verbally and at meetings. Few wanted to express via email their sadness and frustration with a medically-dominated system which, they felt, was using the COVID crisis to lay down rules of exclusion, and which, they intuited, the physicians had always wanted. Midwives with privileges at several other hospitals were being rendered non-essential for cesarean sections, whereas before, they could be there to receive the baby, remaining the “baby doctor” after the cesarean. At one hospital, this restriction was enforced almost until the end of the 2020, when it was lifted.

At the Almonte Hospital, about 45 min from Ottawa, the physicians had decided to request (originally interpreted as “require”) that all birthing patients have an epidural on admission as there was fear about being able to don the PPE in convenient time. The midwives and their clients protested, and it was then clarified that the epidural imposition was not mandatory. But it *was* an indication that physicians and hospital administration can impose control and make decisions based on their own convenience—unless someone blows the whistle.

Then at the second hospital where I have privileges (but not to do vaginal breeches as I can at the Montfort), I was frustrated that the development of an interdisciplinary breech squad had been put off yet again. As a result, there has been an increase in women being designated to a cesarean for breech among midwifery clients through the required physician consult at that hospital.

### Vulnerable Populations in Eastern Ontario

Among the most egregious concerns discussed by the midwives have been the effects of COVID on vulnerable populations. For families needing child protective services, access visits to see children have been canceled. Northern Inuit women who come South for their births to Ottawa have to quarantine for 2 weeks with their babies under security guard before flying back home.

The first report about our numbers came out in June 2020 through the “BORN” system, a database capturing all of the births of registered practitioners in the province^24^. From March 1 to May 29, 2020, there were only 36 COVID-19 cases in pregnant clients that had actually been reported from the participating practices across the entire province^25^. However, by April 23, 2021, with pregnant women accounting for 30% of the patients in the ICU in at least one Toronto hospital, possibly more vulnerable to the new variants new COVID-19 variants (Johnson, 2021), they were placed in the top priority group to receive the vaccine. The extent to which groups are affected—not just by COVID but with regard to their socio/economic/racial/gender distinctions—is currently being missed in our obstetric database. It is complicated but it is starting to be addressed (see BORN, 2016).

#### Moses and the Basket: My Positive and Negative Experiences of Hospital Care During COVID-19 by Candace LeBlanc, a Mother, Doula and La Leche League Leader in Ottawa, Eastern Ontario

I am a Canadian-born woman who was raised in rural Jamaica. I returned to Canada when I was 15 years old, so even though my English has no trace of an accent, I can easily identify what it means to be new to a country and have to learn to understand its culture and customs, in the way that new immigrants do. I also became Muslim when I was 19 and this has also colored the way in which I view and interact with the world. I live with these three ways of life intertwined. I wear a hijab, which I know affects the way people see me. Usually when people hear the name Candace LeBlanc, the last person they expect to see is an English and non-French-speaking hijab-wearing Jamaican Muslim.

I had my first three babies at home with a wonderful Ottawa midwife. The problems with my fourth pregnancy started during COVID lockdown. Living in a low-income neighborhood means that people are extremely fearful and paranoid. I got very ill during the first two trimesters of pregnancy and my children often had the police called on them for riding their bikes outside—an activity that absolutely follows social distancing guidelines, but still police will show up if they are called. So my children had to stay inside all the time because I was not well enough to supervise them outdoors.

During the pregnancy, for circumstances outside my control, I also had to start divorce proceedings and made a decision to initiate a consultation with CAS (Children's Aid Society) to get advice regarding protection for my circumstances. In addition to all this, the children and I got runny noses and because of COVID-19 rules, I couldn't go to my regular midwife visits or get the last blood and urine tests I should have had at the end of the second trimester. I had to wait for several weeks until my and my children's symptoms cleared completely. The day that I could finally go and get the lab tests also happened to be the day that I noticed that my eyes were yellow and I had itchiness all over my body. My midwife asked me about the color of my urine as well. I told her that yes, in fact, my urine had been dark the week before. To my surprise, she said that I should meet her at the hospital right away! Fortunately, I was able to drop the kids off with the community members of my masjid (mosque community) on the way to the hospital.

At the hospital, they found that I had elevated liver enzymes, and recommended that I come in every few days for tests to monitor my levels. A few days later, they found protein in my urine and I was admitted. Thankfully, my mosque community was incredibly supportive and my three children were very well taken care of by many different “aunties”; even groceries were provided. I attribute this to the vision of our masjid, which is not simply to establish another house of worship, but to build a healthy community. It takes a village to raise a child, and my family needed this village more than ever.

Required to remain in hospital for monitoring my high liver enzymes, due to the COVID measures in place at the hospital I was not allowed to have any visitors at all or leave my room for a walk or fresh air. All food or belongings brought for me had to be left downstairs at hospital reception and staff were responsible for bringing them to my room—when and if they had the chance. During two long weeks in the hospital, with nursing all that I had for support, I was struck by the importance of the quality of care. Three nurses stand out who were incredible: a French-Canadian nurse, who also happened to volunteer with La Leche League, a Vietnamese nurse, and a Haitian nurse.

The human touch of *friendship* that these three offered to their professional relationship with me made all the difference. I believe that ethnic background plays a role in this. Other (white) nurses would say, “People cannot drop off food for you because of COVID rules.” Those ever-confusing and evolving rules were applied differently: sometimes my food would show up to my room 3 h later and sometimes it would never come at all. Many nurses refused to heat up my food for me or bring me hot water for tea, citing vague COVID regulations, while my three friendly nurses would take the 5 min to bring my food upstairs, boil me some water for tea, or heat up some food for me—small kindnesses, humanity, when you can't leave your room and really need companionship and empathy. I was thrilled when one of the three ended up being there for the birth—indeed, eternally grateful.

It took two religious communities to help this eventful birth unfold. My grandmother, a white Catholic woman, called upon her church to pray for me and the baby, as did my own masjid community. Through two inductions of my premature baby and 40 h of labor combined, I leaned heavily into these communities for support. Sometimes, it takes a village to *birth* a child.

The Montfort staff were generally respectful of my choices and listened when I explained what I wanted and what my concerns were; they patiently answered my questions and actively engaged with me in my care. This is unique because I have not seen my doula clients treated in this way when birthing in other hospitals across Ottawa. It is possible that this was because, as a doula and a La Leche League leader, and having already given birth myself three times, I am more familiar with birth than the average patient and that the Montfort hospital stands out as actively working with midwives and their clients. And the obstetric/nursing staff there appear more experienced (and therefore receptive) when it comes to an informed choice model of care.

I told my birthing team I wanted to do late cord clamping and allow the baby to hear the Muslim prayers that are traditionally done right after the birth. I had the prayers recorded by my Shaykh on my phone. My doctor, midwife, and nurse allowed me to let my baby listen to them right after the birth and my son was totally calm, breathing well, and resting on my belly. After the prayers, the doctor said, “OK it has been 10 minutes” and my baby was whisked to the warmer. I later learned that the Neonatal Resuscitation Program that all the health professionals in obstetrics take requires transport of the premature baby to the warmer within 30 s, so I was even more grateful and overjoyed that the birthing team had complied with my wishes.

After my son was taken to the nursery, he had breathing issues. This is where things started going awry. A decision was made to send my baby to the Children's Hospital of Eastern Ontario (CHEO) through a rule that dictates that a transfer to the NICU there must be done if the baby is on CPAP >24 h; I was not invited to be a part of this decision, only suddenly informed that the baby would be transported—in the next 30 min. I was told not to worry: although I couldn't travel with him in the ambulance, there would be a bed for me beside him once I arrived at CHEO. I quickly went to my room and packed up all of my belongings and hurriedly got discharged (Thanks again to one of my three heroine nurses!).

When I got back to the nursery, the staff were suddenly acting very strange and whispering to each other. To my surprise, the paper they handed me said that we were going to another NICU at a totally different hospital! They informed me that I was rerouted; later I found out that this rerouting was due to a COVID-19 scare at CHEO. I do wonder whether or not I would have been treated differently if I were white—that is, given more of an explanation as to what was happening with my child.

At the new hospital I was informed, rather dryly, that not only was there no bed for me to stay by my son's side, but if I fell asleep in the chair beside his incubator, I would be asked to leave the NICU. Less than 48 h after giving birth, I had to choose between sleeping and being by my son's side. I could only last for 18 h. I was faced with the decision to place my baby, proverbially, in a basket on the river Nile, and wait for God to bring him back to me, eventually. This is what it felt like to leave my baby in the hospital, alone—or should I say, in the House of Pharaoh—to me, a foreign place, unnatural in its design and oppressive in its rules, a place that was governed by liability, not empathy, a place with too much “head” and not enough “heart.” This was my Red Sea to cross.

After a few days of going back and forth to the hospital, I realized, having used the early labor lounge as a doula, that it was available; I dragged my stuff in there and fell asleep. However, when the staff found out, they locked it so that I couldn't use it, even though it was not being used by anyone else. After 6 long, exhausting days, my son had been off CPAP for a few days and could take a bottle. I therefore felt he was well enough to come home. First, I phoned my midwife to ask her whether she could come and follow up on the baby's health at home if I signed out Against Medical Advice (AMA). We had a discussion about my perception of how I was being treated as a racialized woman and she agreed to support me in doing whatever was best for the baby. Then, I asked the resident whether or not I could take out the baby AMA. The resident told me she would go and call the doctor so that I could consult with him. I waited patiently, but she didn't return for nearly 2 h. Eventually she informed me that she had reported me to CAS, without consulting me first. I felt that she did this because I was Black. I had not even consulted with the doctor yet, nor been told they were calling CAS, which—I checked—is the first step before getting CAS involved.

I called my midwife to ask her to advocate for me to the NICU staff, and when she realized that the nurse manager had been informed by the resident that I already had a CAS file opened up on me, my midwife made sure to inform them, “This is a file that *Candace* opened herself three weeks ago. She is an educated, well-informed La Leche League Leader, and actually, Jamaican and from a group that tend to know their rights and how to manage the system—hence her pro-active opening of the CAS file, to protect her and her family.”

My goal was to do what was best for the baby, whether it was to keep him in or take him out of the hospital. The CAS file was closed and, a day or so later, they agreed to move me to CHEO. It was heaven; I could finally have a bed next to my baby.

Unfortunately, my first night at CHEO, my baby and I shared a room with a young mother with only a curtain between us. Thus, I overheard that she also wanted to take her baby out of the hospital AMA. Late into the night she could be heard yelling at the doctors and nurses and being extremely disrespectful to them. I overheard her say to her partner on the phone that she had a previous police record, was not legally allowed to be within a certain distance of her current partner, and worried that the police would charge her for that offense if they were to be seen together. She made disturbing jokes about her infant falling off the couch while she was on the phone but insisted to the doctors she “just didn't know” what had happened to her son. Her baby had a broken humerus and CAS suspected child abuse. But she told her CAS worker that she wanted to leave the hospital that night.

“There is no way she is leaving tonight” I thought to myself.

As she continued her rude behavior toward the staff, I learned her baby was exactly the same age as mine, 2 weeks old. The staff weakly tried to convince her to stay overnight. She flatly refused and signed her baby out AMA. She left with her baby that night. She was white.

**Figure 4:**
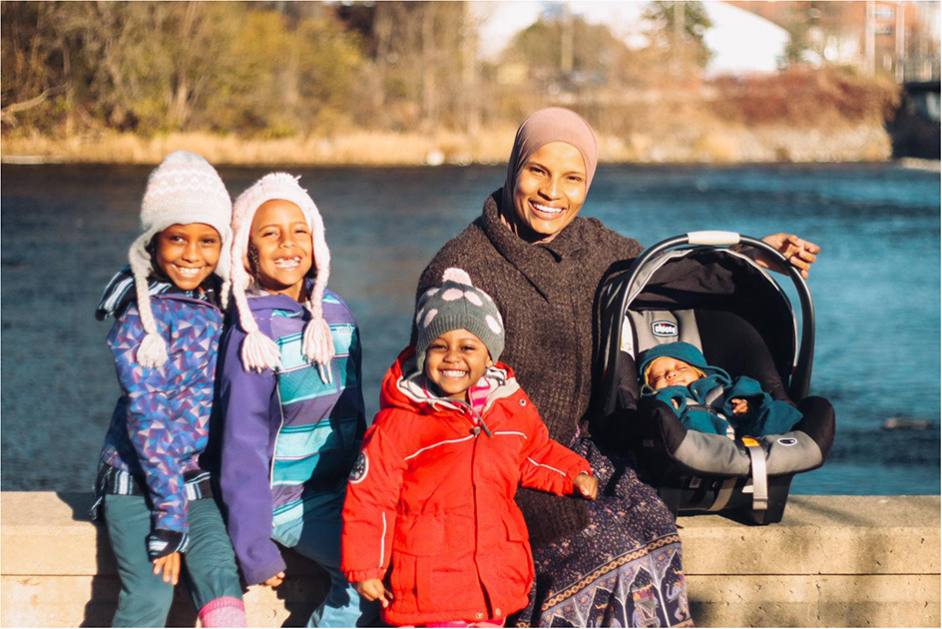
Candace and her family after crossing over the hospital Red Sea of tape. Photo by Kamal Abdulhakim. Used with permission.

I crossed the Red Sea of racism and inconsistent protocols and got to take my son out of hospital and bring him home to meet his sisters 2½ weeks after his birth. I was permitted under the proviso that my midwife visit me a couple of times a week, which she did faithfully. He is now a healthy growing boy who indeed came back to his people in a blessed way, just like Moses (see Figure 4).

#### Summary and Analysis of Candace's Story

While Candace has a low economic status—in particular because she married young and now is a single mother with four children—she has some university education, has a white father, and is well aware of white middle class biases and people's unknown or unspoken attitudes. We would therefore be hard-pressed to suggest she is of the proverbial “low” socio-economic class, which inherently also can infer less education. In her story, she perceives the subtle racism so frequently spoken about among the IBPOC population—how she was treated differently by the nurses who respected her in one hospital and by a staff in a different hospital, following her desperate situation with nowhere to sleep. Ironically, a staff member injudiciously called CAS prematurely, without telling her or honoring her wishes to speak with the physician, making assumptions until they found out that it was Candace who had opened up her own file. And the final blow: how she could only interpret the treatment of a white woman, acting irresponsibly yet being discharged anyway, as a sign of white privilege.

In the next section, we explore some inherent gaps in the population that midwives serve in Ontario, looking at the demographics of midwifery clients by socio-economic classes designated by neighborhood.

#### Data on Socio Economic Status of Midwives' Clients

While COVID-19 was waging, a retrospective cohort study emerged in our national midwifery journal about a sobering concern that has troubled the midwifery profession in Ontario. Prior to midwifery legislation in Ontario in 1993, because Canadian health insurance covered only hospital births with physicians and the far North, largely only people who could afford the full cost of midwives hired them. Many midwives would take refugees, immigrants, lower income clients, and those living rurally at a reduced rate, or barter for lambs, plants, pottery, bread, carpentry, and child care. For many midwives, the impetus for legislation was to be able to serve the population that could not afford midwives (Daviss, 1999).

Fast forward, a new study based on neighborhood-level maternity care 2006–2017 demonstrates that one or two decades after legislation, childbearers of low socio-economic status in Ontario are less likely to receive midwifery care than those of high socio-economic status. The researchers were careful to say that ethnicity was not part of their study (Darling et al., 2020). Fortunately, the rest of the articles in this edition of the *Canadian Journal of Midwifery* provide models that are stepping up to the plate to change the situation.

Given Candace's example, we have some difficulty in assumptions made about what is meant by the term “low socio-economic class,” since Candace, living in a low-income neighborhood, would have been classified as such in this study, not particularly accurately. On reflection, she suggests that it invites assumptions—evident in the hospital staff's attitudes toward her—as “uneducated, ignorant reactive and uninformed,” in her case, she felt, because she was Black and wearing a hijab. The fact that midwives do not well serve the populations living in those neighborhoods is disturbing, but the stereotyping can also serve to revictimize the people living there.

### Data Demonstrating an Increase in Home Births in Ontario With COVID-19

The data in Table 2 were taken from the BORN database [BORN (Birth Outcomes Registry Network) Ontario, 2020], but preliminary because the data were not all yet incorporated into the database for the 2020 months at the time of retrieval, November 8, 2020. It shows that in 2019, the planned hospital birth rate among midwifery clients each month was approximately 80–82%, and the planned homebirth rate was 13–14%. By May of 2020, the trend for this preliminary data indicated that the planned hospital birth rate had dropped to 74.4%, and the planned home birth rate had climbed to 20.0%^26^.

### Summary Section How COVID-19 Has Affected Maternity Care in Eastern Ontario

This section has demonstrated the stressors faced by the midwives as a result of COVID-19. It has revealed some goodwill, responsibilities and ethics both of the midwives and hospital management, but it has also exposed some of the opportunistic reactions of care providers and authorities. It raises the concerns about the subtle racism and the reality that childbearers of low socio-economic status in Ontario are less likely to receive midwifery care than those of high socio-economic status.

We move next to the story of Guatemala, where Indigenous oppression is not quite so subtle and professed universal health care not as readily available.

## Guatemala: Experiences Among the Traditional Midwives of Guatemala

### Stepping Up to the Plate on Gender Equality

Working with the healthcare providers in Totonicapán, the MNCH program implemented by Horizons and PIES was very careful to include programs that engaged the larger issues of lack of resources and violence in the communities they were serving prior to COVID-19. They carried out workshops not just on midwifery skill sharing and childbirth but also on gender equality. In addition, they have been able to provide personal counseling sessions for both women and men in that regard. One of the stories, told through a midwife's eyes, demonstrates how cultural attitudes devalue women, and may affect long-term health outcomes:

Valenzuela grew up watching her father violently assault her mother. His attacks were both verbal and physical, often telling Valenzuela and her mother that they were useless women. This violence resulted in Valenzuela's mother experiencing several miscarriages. It was seeing her mother experience this loss at the hands of her father that marked her for life. This story captures how gender-based violence has resulted in significant consequences for the well-being of women and their children in Totonicapán (Horizons, 2018).

**Figure 5:**
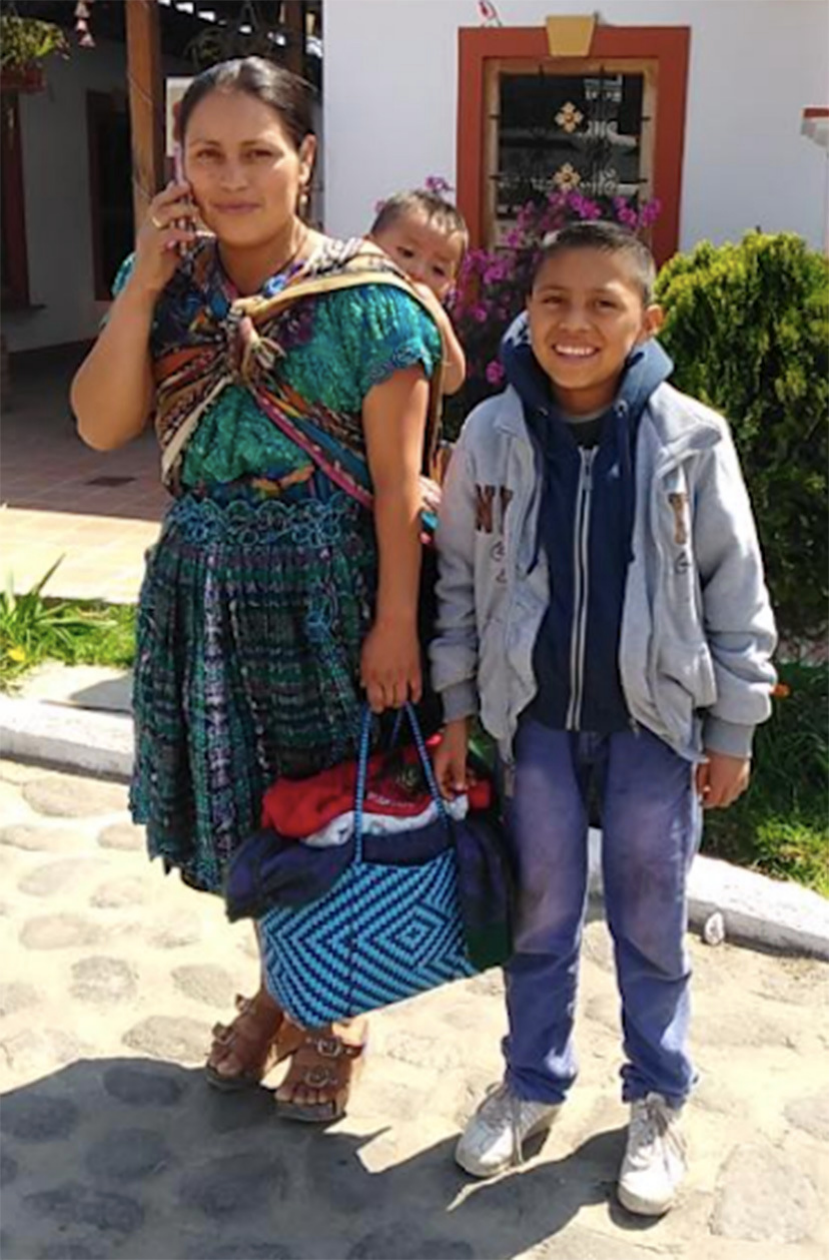
Valenzuela Cos Matul on her cellphone, her children in tow, representing the new generation of comadronas, teaching best practices of maternal child health, the rights of women, and the impacts of gender-based violence. Photo by Betty-Anne Daviss in Totonicapán, 2017. Used with permission.

However, today, Valenzuela Cos Matul is a *comadrona*, has become a leader in her community and, has helped train other midwives on best practices for providing maternal and child health care (see Figure 5). As part of the project, more than 940 midwives received training in safe birthing practices and care, and also on the impact of gender-based violence and the need to respect and recognize the rights of women.

### How COVID-19 Affected the Midwives in Totonicapán

#### Introducing Iris Champet

**Figure 6:**
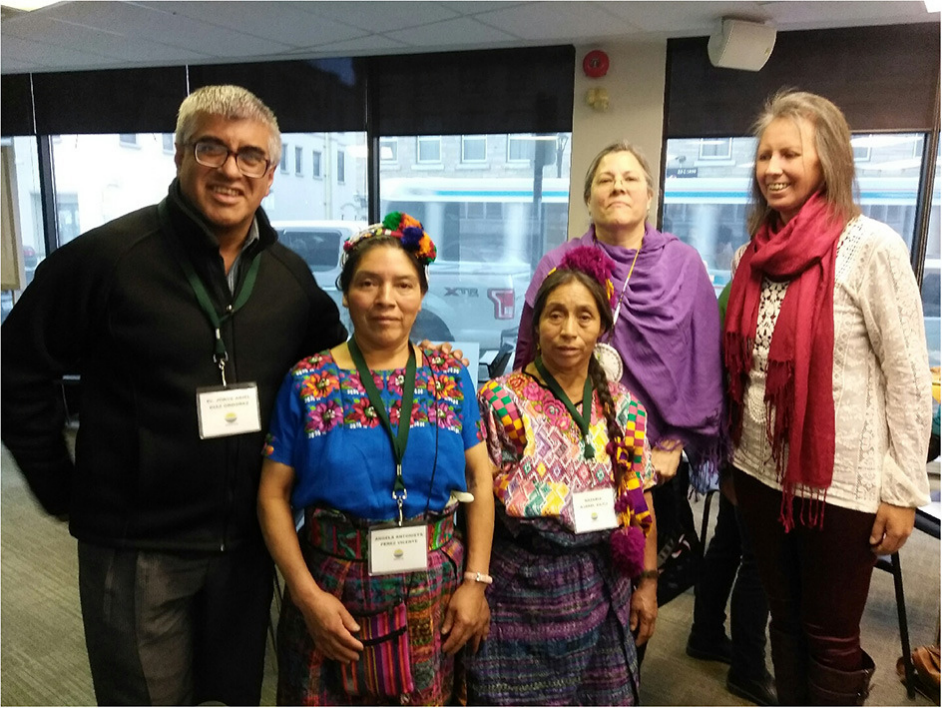
From left to right: Dr. Diaz, Angela Antonietta Perez Vicente and Nazaria Ajamel Xiloj from Momostenango, Guatemala, with Dorothy Green from Kenhte:ke Midwives Tyendinaga Mohawk Territory (in purple shawl) and Lynn Brant, nurse practitioner and community advocate—two Mohawk women offering support for traditional Indigenous knowledge exchange. Photo in Kingston, Ontario provided by the Horizons' Office (2017), used with permission.

Dr. Iris Champet was born and raised in Totonicapán, educated as a primary school teacher, physician, and surgeon, and has worked for approximately 10 years supporting the work of midwives, health promoters, health commissions, and community leaders. She coordinates the MNCH project for PIES. She and Betty-Anne met in Guatemala in 2017 when the first contingency of Canadian health care providers—doctors, nurses, and midwives—was sent to Guatemala for the PIES/HORIZONS project. Betty-Anne requested that PIES/HORIZONS create a chapter for a book she was writing, Birthing Models on the Human Rights Frontier. Although it did not transpire, Dr. Champet provided some details for the book about the work the comadronas were doing to seek remuneration for their work. That is, although a bill to compensate them had been passed by Congress it was vetoed by the President of Guatemala (Daviss, 2021). For this current article, Dr. Champet sent the following account in Spanish, which Betty-Anne translated. See Figure 6 for a photo of the Guatemalan team meeting with the Indigenous team in Kingston, Ontario.

#### COVID-10 in Totonicapán

The COVID 19 pandemic affected all of us. In particular, the traditional midwives, called *comadronas*, found themselves in need of making changes in their personal and family lives and in the development of their activities as midwives. On March 13, 2020, the first positive case was detected in Guatemala, and since then, various directives have been given by the President. There were no longer knowledge-exchange meetings with the comadronas, because crowds had to be avoided. Then a “curfew” was decreed, a restriction of mobility from 4:00 p.m. to 4:00 a.m. every day. If you circulated during these restricted hours, there was a fine to pay.

With the curfew, it was not clarified whether or not one was allowed out to deal with emergencies. This made it difficult for the midwives to work, because as is well known, the greatest number of deliveries are attended at night. Thus, several comadronas chose to commute to their patient's home at permitted times to avoid paying the fine; if the labor did not develop, they returned to their homes in the morning and returned again to the pregnant woman's house in the afternoon until she went into labor.

Another downside was that in the health services, there was so much attention paid to COVID-19 that the midwives had less support. Pre-COVID, the comadronas were allowed to go to the hospital with their mothers in labor, explain the reason for the transfer, and usually even do the delivery. (Others were not permitted to accompany the mothers). Currently, the comadronas are not permitted to enter nor attend the deliveries because of the protocols that exist for the prevention of COVID-19.

Yet another change is that women are choosing not to go to hospitals for fear of getting infected. This has meant an increase in the care of pregnant, parturient and puerperal women by traditional midwives, both in Totonicapán and at the national level. This has posed difficulties with births that are complicated and *need* referral. The comadronas have been trained to identify danger signs, but have found that women and their families do not always accept referrals in these cases, both for fear of hospital contagion and because misinformation abounds in the community; there are rumors that you have to get swabbed for COVID-19 if you go to the hospital, when in fact that is only if you have signs and symptoms. The mothers are also afraid that if they test positive, their baby will be taken away from them.

On many occasions, the comadronas have increased expenses, because they have to depend on their own resources as they try to figure out how to return to their homes. They are not permitted to be in the hospital with their patients, and the cars that take the patients do not wait for the midwives. *There is no public transportation, and the comadronas do not own their own cars*. Even before COVID-19, these midwives lacked appropriate equipment for the care of women and their families. They have cloth masks, donated by the Maternal and Neonatal Health Project, but they are not N95s. And they do have direct contact with people.

At the beginning of the restrictions, as the directive was to “stay at home,” the community authorities were very demanding during hospital transports: the midwives had to be duly identified with their midwives' card, and sometimes they had to pass through review of their documents by authorities up to five times on the way to the hospital.

Another difficulty has been figuring out a system by which to make the restrictions known. After identifying some of the problems, the President of Guatemala clarified that maternal and vaccination care should continue on a regular basis. In addition, the Indigenous Peoples' Unit issued a circular, which was sent up to the authorities, indicating that the mobilization restriction did not apply to midwives and that they should be supported in their work. This strong level of support gave them more confidence.

Within the MNCH Project, funded through Horizons of Friendship and the Government of Canada, liquid soap, cloth masks, waterproof coats, protective glasses, hats, towels, and gloves, among others, were donated to the midwives; they have been a tremendous support. The MSPAS (Ministerio de Salud Pública y Asistencia Social, 2020) has donated some supplies that have been distributed to some midwives; it has not been possible to reach all of them. These comadronas are very afraid of becoming infected, because they do not know who is and who is not infected with COVID 19, as little testing is available.

#### Ishim Yac

Ishim Yac, a young woman who has been involved with the comadronas, works for the Guatemalan Stove Project, an NGO in a Neighboring area to Totonicapán—Quetzaltenango. She sent us the information below about the general situation across the region in February 2021 (translated by Betty-Anne):

COVID-19 has demonstrated how precarious our health and education system are, as well as our entire infrastructure. When COVID-19 hit our country, the country was closed. This included markets, public transport, schools, and businesses, etc. as a form of restraint, following the installation of temporary hospitals for COVID-19. Unfortunately, there was no equipment, human resources, and above all, unoccupied space or beds to care for COVID-19 patients.

The closure of the country definitely affected the poor population, mostly Indigenous people. In Totonicapán, the women mostly deal with middlemen who sell their *huipiles* (colorful embroidered blouses) in the market, but with the market closed, they were unable to work. This has affected the economy…People have chosen to cure themselves of COVID-19 with natural treatments such as eucalyptus, ginger, lemon, and chamomile rather than go to hospital.

COVID-19 cases in rural areas are very low compared to large cities, such as Guatemala and Quetzaltenango, where the numbers are high. As for education, children can't attend school and distance education has become much more complicated in an environment where parents can't read and write. You can imagine how complicated it is where virtual education is almost impossible, and children don't have a device to zoom in on, meet teachers or each other, to receive classes.

Strong hugs from afar.

#### Summary of Section Guatemala: Experiences Among the Traditional Midwives of Guatemala on Totonicapán, Making Some Comparisons With Ontario

The Guatemalan comadronas demonstrate through this scenario how resilient and flexible they are. Aggravated by the recent breakdown of the economy, they are so dedicated and responsible to the mothers with whom they work that they sleep overnight at women's houses to make sure they are not caught by the curfew. No doubt they were stopped many times on the road by police because of their Indigenous status. Unlike Ontario, where people of lower socio-economic status (SES) are less likely to receive midwifery care than people of higher SES, in Guatemala, indigenous women are served by midwives from their communities, who are in the same SES group. Tammy in Northern Ontario, serving the Amish/Indigenous population, echoes the feeling of isolation of the traditional midwives during COVID-19 but appears to feel more appreciated and supported by authorities for her situation.

In both countries, midwives' roles in hospital or with local authorities, and thus client care, appeared to suffer with COVID-19 as better systems were being worked out. The Canadian midwives could learn from the PIES program about how to face gender-based violence. The upside in both countries is that midwives are clearly becoming the link for seeking choice in their communities, and childbearers are increasingly discovering the benefits of staying home for their births. It was a positive development that midwives in both Ontario and Guatemala were granted the right to have a vaccination in the first phase of vaccinations in March, 2021, a recognition of their vulnerability. In Guatemala, the comadronas were slated for their vaccination ahead of the firemen and paramedics^27^.

With increased clientele because of COVID-19, and recognition of their roles by the government through the vaccination program, one would think that the comadronas' contribution and faithfulness to their community could be rewarded by the small wage for they are asking, from their national government.

## Conclusions: Peeling Off the Masks

We can now state that a comparison about pandemic effects between a province in a high resource country (Canada) and a low resource country (Guatemala) reveals similar problems and similar solutions. The first outstanding similarity is that Indigenous populations suffer marginalization in both Totonicapán and Ontario. The femicide problem is of increased concern in both countries for Indigenous peoples during COVID-19, with violence proven to be spiking in Canada, with suspicions of the same thing happening in Guatemala, although the problem is clearly affecting women across Guatemala as a whole. The health conditions of people on the Indigenous reservations in Ontario, with contaminated or threatened water supplies, now additionally suffering under lockdown, are also somewhat similar to those in Guatemala.

As a result of COVID itself or the fear of restrictions and interventions, home births are on the rise—even with those who are higher risk and need to be in hospital—in both jurisdictions. The obvious upside in Ontario is that first, midwives get paid, and second, the Federal government has increased funding to Indigenous midwives. In Totonicapán, where PIES is recognizing more than ever the fortitude and value of their traditional midwives, the national government should now find it harder to ignore the will of the people who are increasingly demanding these midwives' services. Whether or not the government will be fair about it and agree to pay them—thereby reducing the strain on the hospital system—remains to be seen.

Just as the white privileged US population tends to have polarized attitudes toward its IBPOC population, Guatemalan authorities and *Ladino* (non-Indigenous people of mixed origin, similar to the *mestizos* of Mexico) peoples have had an historically polarized dissonance toward their Indigenous peoples. In Canada, despite our national identity of making sure that “nobody is left behind” because the vulnerable are part of “us,” the issues of racialized people have still been largely rendered invisible, ethnically diverse neighborhoods have been hit harder by COVID-19, with substantial proof from the Black population. Subtle racism continues in health care. Indigenous, 2SLGBTQQIA and women's communities continue to struggle.

Midwives, nurses, and physicians working with vulnerable populations may see, but do not always work on, rectifying the inequities, the biases, and the oversights. Midwives also fall prey to being abused themselves, as they try to mitigate a medical system that does not always share their values of informed choice. This can become more abusive as higher echelons take advantage of the pandemic to impose restrictions they have often wanted even in non-COVID times. However, seeing inequities laid bare and how far authoritarianism can go without whistle blowers can be turned into an opportunity. The mirror that is held up during these times gives authorities a chance to consider rights, such as they did with regard to rights of babies in the nursery at the Montfort, and they did admit being “wrong” in phoning CAS on a Jamaican hijab-wearing patient a little too quickly—before telling her.

When hospitals excuse themselves for not stepping up to other reforms, such as making the change to allow vaginal breeches attended by midwives, “because of COVID-19,” the pandemic becomes a crutch to beg forgiveness, rather than a tool to make the changes needed.

We offer this work as a means to understand the problems and articulate how to improve our systems that are inherently racist, colonialist, sexist, white cisnormative, and biased toward a medical hegemony. We suggest that one way to make our health care systems safer and more culturally appropriate would be to enhance community-based midwifery practices and collaborative models (Daviss, 2021).

If we ever get to take off our COVID-19 masks, it would be good to peel off the larger whitewashed masks that have been rendering the issues of racism and marginalization of midwives not an issue that is supposed to be discussed in Canada. As with activists in other arenas such as the environment, as Ontario health care advocates, we hold up hope that COVID-19 can be the catalyst that challenges the standard narrative, exposing and then eliminating the old ruts and blind spots of inequality and discrimination that until COVID-19, our obstetric and hospital and health care system hierarchies and our white citizens were managing to put on the backburner.

## Data Availability Statement

The raw data supporting the conclusions of this article will be made available by the authors, without undue reservation.

## Ethics Statement

Ethical review and approval was not required for the study on human participants in accordance with the local legislation and institutional requirements. The patients/participants provided their written informed consent to participate in this study. Written informed consent was obtained from the individual(s) for the publication of any potentially identifiable images or data included in this article.

## Author Contributions

B-AD oversaw the development of the article and encouraged community input. TR wrote her experience in her Indigenous registered midwife practice in Northern Canada. CL wrote the section about her first birth in hospital and the subte racism she felt. IC wrote the section on Guatemala. BB advised B-AD during the development of the paper, provided useful input, and editing throughout. LG provided insight and facilitated the exchange on the situation on Guatemala during COVID-19. AA wrote part of and consulted over the Indigenous history in Canada. All authors contributed to the article and approved the submitted version.

## Conflict of Interest

The authors declare that the research was conducted in the absence of any commercial or financial relationships that could be construed as a potential conflict of interest.

## Footnotes

^1^OHIP: Ontario Health Insurance Card which all Ontario residents are entitled to and which pays for all of their health care, including all perinatal care, excluding only dentistry, pharmaceuticals, and extraordinary luxuries like *in-vitro* fertilization.

^2^https://www.indexmundi.com/guatemala/maternal_mortality_rate.html

^3^https://www.indexmundi.com/canada/maternal_mortality_rate.html

^4^https://www.usaid.gov/sites/default/files/documents/1864/Guatemala-Nutrition-Profile-Mar2018-508.pdf

^5^IBPOC: Indigenous, Black, and People of Color.

^6^As part of the project, an initiative funded by Global Affairs Canada, Canada-Guatemala knowledge exchanges were set up by PIES de Occidente (The Association for Health Promotion, Research, and Education) and Horizons of Friendship, a Canadian international development organization.

^7^http://www.thedialogue.org/wp-content/uploads/2017/02/Educational-Challenges-in-Guatemala-and-Consequences-for-Human-Capital-and-Development.pdf

^8^2SLGBTQQIA stands for 2Spirit, Lesbian, Gay, Bisexual, Transgender, Queer, Questioning, Intersex, and Asexual. Available online at: https://www.mmiwg-ffada.ca/wp-content/uploads/2019/06/Final_Report_Vol_1a-1.pdf.

^9^The World Health Organization, International Confederation of Midwives, and International Federation of Gynecology and Obstetrics describe a *skilled attendant* is an accredited health professional—such as a midwife, doctor, or nurse—who has been educated and trained to proficiency in the skills needed to manage normal (uncomplicated) pregnancies, childbirth, and the immediate postnatal period, and in the identification, management and referral of complications in women and newborns (World Bank, 2018 p. 1). This definition excludes traditional midwives. In Daviss and Davis-Floyd (2021) we seek acknowledgment of the tireless and fearless expertise they often provide for their communities, and of the need to integrate them into health care services instead of eliminating them.

^10^https://www.blackclassaction.ca/plaintiffs

^11^https://d2071andvip0wj.cloudfront.net/totonicapan-tension-in-guatemalas-indigenous-hinterland.pdf

^12^The murder of women, committed by men, for the simple reason of their being women (http://www.oea.org/es/mesecvi/docs/DeclaracionFemicidio-EN.pdf).

^13^https://www.theguardian.com/healthcare-network/2018/mar/07/health-workers-stop-thousand-women-killed-guatemala-femicide and https://www.insightcrime.org/news/analysis/why-does-latin-america-have-the-world-s-highest-female-murder-rates/.

^14^http://ggm.org.gt/wp-content/uploads/2019/06/Estad%C3%ADsticas-mayo-y-acumulado-2019-CAIMUS-GGM.pdf

^15^https://d2071andvip0wj.cloudfront.net/totonicapan-tension-in-guatemalas-indigenous-hinterland.pdf

^16^https://briarpatchmagazine.com/articles/view/reviving-indigenous-authorities-in-guatemala

^17^https://www.ontariomidwives.ca/indigenous-midwifery

^18^https://www.ontariomidwives.ca/self-determination-key-improved-outcomes-midwifery-care-within-torontos-indigenous-community

^19^https://outlookseries.com/A0996/Science/3989_Michele_Gelfand_University_Maryland_Understanding_Tight_Versus_Loose_Cultures_Critical_Interdependent_World_Michele_Gelfand.htm (accessed February 13, 2021).

^20^http://www.algomapublichealth.com/disease-and-illness/infectious-diseases/novel-coronavirus/current-status-covid-19/#NO

^21^https://www.publichealthontario.ca/-/media/documents/ncov/epi/2020/06/covid-19-epi-diversity.pdf?la=en

^22^https://www.aljazeera.com/news/2020/4/16/fears-grip-guatemalas-indigenous-groups-as-coronavirus-sets-in

^23^https://www.obgyn.utoronto.ca/sites/default/files/jon_barrett.pdf

^24^Midwives cannot get paid unless they turn in their data on the births they attend, and to add in their COVID cases would not be too much of a stretch.

^25^https://www.bornontario.ca/en/index.aspx

^26^The interpretation and conclusions contained herein do not necessarily represent those of BORN Ontario.

^27^https://www.mspas.gob.gt/noticias/noticias-ultimas/5-noticias-mspas/1214-inicia-distribución-de-vacuna-covid19-astrazeneca-covishield-a-las-29-áreas-de-salud.html; https://covid-19.ontario.ca/getting-covid-19-vaccine-ontario
